# Genotypes and public health potential of *Enterocytozoon bieneusi* and *Giardia duodenalis* in crab-eating macaques

**DOI:** 10.1186/s13071-019-3511-y

**Published:** 2019-05-22

**Authors:** Li Chen, Jianguo Zhao, Na Li, Yaqiong Guo, Yuanyuan Feng, Yaoyu Feng, Lihua Xiao

**Affiliations:** 1 State Key Laboratory of Bioreactor Engineering, School of Resource and Environmental, East China University of Science and Technology, Shanghai, 200237 China; 2 Laboratory of Tropical Veterinary Medicine and Vector Biology, Institute of Tropical Agriculture and Forestry, Hainan University, Haikou, 570228 Hainan China; 3 College of Veterinary Medicine, South China Agricultural University, Guangzhou, 510642 China

**Keywords:** *Enterocytozoon bieneusi*, *Giardia duodenalis*, Genotypes, Multi-locus genotyping, Crab-eating macaques

## Abstract

**Background:**

*Enterocytozoon bieneusi* and *Giardia duodenalis* are common human and animal pathogens. Studies have increasingly shown that non-human primates (NHPs) are common hosts of these two zoonotic parasites. However, few studies have explored the genetic diversity and public health potential of these pathogens in laboratory monkeys. In this study, we examined the genetic diversity of the two pathogens in crab-eating macaques (*Macaca fascicularis*) in a commercial facility in Hainan, China.

**Results:**

*Enterocytozoon bieneusi* and *G. duodenalis* were detected by PCR analysis in 461/1452 (31.7%) and 469/1452 (32.3%) fecal specimens from the animals, respectively. Significantly higher detection rates of *E. bieneusi* were detected in males (36.5%, 258/706) than in females (26.7%, 160/599; *χ*^2^ = 14.391, *P* = 0.0001), in animals with loose stools (41.4%, 151/365) than those with normal stool (28.5%, 310/1087; *χ*^2^ = 20.83, *P* < 0.0001), and in animals of over 3 years of age (38.6%, 135/350) than those of 1–3 years (29.6%, 326/1,102; *χ*^2^ = 9.90, *P* = 0.0016). For *G. duodenalis*, the detection rate in males (33.4%, 236/706) was higher than in females but not statistically significant (30.2%, 181/599; *χ*^2^ = 1.54, *P* = 0.2152), in monkeys with loose stools (41.1%, 150/365) than those with normal stools (29.3%, 319/1087; *χ*^2^ = 17.25, *P* < 0.0001), and in monkeys of 1–3 years of age (36.6%, 403/1102) than those over 3 years (18.9%, 66/350; *χ*^2^ = 38.11, *P* < 0.0001). Nine *E. bieneusi* genotypes were detected in this study by DNA sequence analysis of the internal transcribed spacer of the rRNA gene, namely Type IV (236/461), Peru8 (42/461), Pongo2 (27/461), Peru11 (12/461), D (4/461) and PigEbITS7 (1/461) previously seen in NHPs as well as humans, and CM1 (119/461), CM2 (17/461) and CM3 (3/461) that had been only detected in NHPs. DNA sequence analyses of the *tpi*, *gdh* and *bg* loci identified all *G. duodenalis* specimens as having assemblage B. Altogether, eight (4 known and 4 new), seven (6 known and 1 new) and seven (4 known and 3 new) subtypes were seen at the *tpi*, *gdh* and *bg* loci, leading to the detection of 53 multi-locus genotypes (MLG-B-hn01 to MLG-B-hn53). Most of them were genetically related to those previously seen in common Old-World monkeys.

**Conclusions:**

Data from this study indicate a common occurrence of zoonotic genotypes of *E. bieneusi* and assemblage B of *G. duodenalis* in farmed crab-eating macaques in Hainan, China.

## Background

*Giardia duodenalis* and *Enterocytozoon bieneusi* are common human pathogens. At present, there are more than 200 million of annual giardiasis cases in humans, while microsporidiosis is a common cause of diarrhea [1, 2]. The incidence of giardiasis has been reported to be 5.5 per 100,000 people in the UK and 7.5 per 100,000 people in the USA [3]. In China, almost 30 million people are infected with *G. duodenalis* every year [2]. For *E. bieneusi*, the detection rates range between 2–78% in industrialized and developing countries [4–6]. Over 200 giardiasis outbreaks have been reported in the world during the period 2004–2016, while *E. bieneusi* also caused an outbreak in France [6–9].

Non-human primates (NHPs) are important experimental animals in public health research because of their high genetic similarity to humans [10]. A growing number of studies have found that NHPs are the hosts of many parasites, including gastrointestinal protists *E. bieneusi* and *G. duodenalis*, which are transmitted in similar fecal-oral routes [2, 11]. In addition to affecting the health and wellbeing of these laboratory animals, both pathogens are potentially zoonotic, causing diseases in humans [12, 13].

Of the 17 known human-pathogenic microsporidian species, *E. bieneusi* is the most common [14]. Based on sequence analysis of the internal transcribed spacer (ITS) of the rRNA gene, more than 200 *E. bieneusi* genotypes have been identified [15]. In phylogenetic analysis, these *E. bieneusi* genotypes are divided into at least 11 groups [16]. Among them, Group 1 contains most genotypes found in humans and many genotypes in animals, thus is considered to be the zoonotic group. In contrast, Groups 2–11 include genotypes found in specific groups of hosts, including humans, thus are considered more host-specific. There is also a so-called outlier group in dogs [17–22]. Thus, genotyping *E. bieneusi* in NHPs can help us understand the zoonotic potential of *E. bieneusi* in these animals.

At present, more than 50 *E. bieneusi* genotypes have been found in NHPs, most of which belong to Group 1 [23]. Among them, genotypes A, D, Type IV, EbpC, Peru7, Peru8, Peru11, PigEBITS7, Henan-V, WL15, I and BEB6 have been found in humans in several countries, including China [6, 13, 15, 18, 24–30]. Of these, genotypes A and I appear to be more common in diarrheic children in China than other genotypes, suggesting that there could be differences in infectivity or virulence among *E. bieneusi* genotypes [6, 31]. Therefore, NHPs are potential reservoir hosts for zoonotic transmission of *E. bieneusi*.

Similarly, eight distinct *G. duodenalis* assemblages (A-H) have been identified by genetic analysis of triosephosphate isomerase (*tpi*), ssrRNA, β-giardin (*bg*), glutamate dehydrogenase (*gdh*) and other genes [2, 32, 33]. Among them, assemblages A and B are most commonly found in humans and NHPs, assemblages C and D are mainly detected in canines, assemblage E mainly infects ruminants and other hoofed animals, whereas assemblages F, G and H usually infect cats, rodents and seals, respectively [2]. Similar to *E. bieneusi*, genotyping *G. duodenalis* also can help us to understand the transmission of this pathogen.

NHPs are also potential reservoir hosts for zoonotic transmission of *G. duodenalis* [2, 12]. In previous studies, assemblages A, B and E have been identified in humans and NHPs [34]. Among them, assemblage B appears to be most common, while assemblage E is only occasionally detected [34–38]. Although assemblage A has been further classified into three major sub-assemblages (AI–AIII) by sequence analysis of several genetic loci, consistent secondary classification of assemblage B has not been established [2, 12, 39, 40]. Multilocus genotyping (MLG) has been used in several studies to understand the host specificity and zoonotic potential of assemblage B in human and NHPs [41–44]. Controversies exist on the differences in virulence between assemblages A and B in humans [45]. There are no such studies on *G. duodenalis* in NHPs.

In the present study, we examined the prevalence of *E. bieneusi* and *G. duodenalis* in fecal specimens from commercial crab-eating macaques (*Macaca fascicularis*) in Hainan. The genetic diversity of the two pathogen species was assessed using sequence analysis of the ITS (*E. bieneusi*) and using MLG analysis of the *tpi*, *gdh* and *bg* gene (*G. duodenalis*). The data generated were used to explore the human-infective potential of these two common gastrointestinal parasites in NHPs.

## Methods

### Specimen collection

A total of 1452 fecal specimens were collected in April 2016, June 2017, October 2017 and January 2018 from laboratory crab-eating macaques kept on a commercial farm. The farm was founded in 2003 and has been awarded full accreditation from the International Association for Assessment and Accreditation of Laboratory Animal Care (AAALAC) since 2008, with over 20,000 animals at the sampling time. All animals were kept in separate cages (1 × 1 × 1 m) after they were born on the farm, with approximately 30 animals kept in each room of ~ 60 m^2^. The cages were elevated 1 m from the ground so that the feces could fall onto the ground. Every monkey had contact with animals in the neighboring cages. The rooms were cleaned every morning and afternoon to ensure a clean living environment. Feed, fruits (apple, banana and peach) and drinking water were regularly distributed by farm staff to each cage every day.

The sampling plan took into consideration the number, age and sex of animals on the farm, and the needed number of positive specimens to generate data for a meaningful assessment of the distribution and human-infective potential of *E. bieneusi* and *G. duodenalis* genotypes in these animals. Among the sampled animals, 706 were male, 599 were female and 147 sampled animals had missing information on the sex. The sampled animals belonged to two age groups: 1102 were 1–3 years-old and 350 were adult monkeys older than 3 years. Regarding the latter, as monkeys over 4 years-old were often sold, the oldest animals sampled in the study were 5 years-old. At the time of sampling, 365 monkeys had loose stools, as defined by runny fecal consistency, and 1087 monkeys were apparently normal. The specimens were stored in 2.5% potassium dichromate solution at 4 °C prior to DNA extraction.

### DNA extraction

The stored fecal specimens were washed three times with distilled water by centrifugation at 2000×*g* for 10 min. Genomic DNA was extracted from the washed fecal material using the FastDNA SPIN Kit for soil (MP Biomedicals, Santa Ana, CA) [46]. DNA was stored at − 20 °C before being used in PCR analysis within one year.

### Detection and genotyping of *Enterocytozoon bieneusi* and *Giardia duodenalis*

*Enterocytozoon bieneusi* was detected by nested PCR amplification of a 392-bp fragment of the rRNA gene containing the entire ITS sequence [47]. The genotypes of *E. bieneusi* found in this study were determined by sequencing the PCR products and comparing the sequences obtained from the specimens with the reference sequences from known genotypes. The established genotype nomenclature was used in naming *E. bieneusi* genotypes identified in this study [15].

*Giardia duodenalis* was detected by nested PCR amplification of a 530-bp fragment of the *tpi* gene, a 511-bp fragment of the *bg* gene and a 599-bp fragment of the *gdh* gene [48–50]. The specimen was considered *G. duodenalis*-positive if any of the PCRs generated the expected PCR product. The genotypes of *G. duodenalis* found in this study were identified by sequencing PCR products from *G. duodenalis*-positive specimens and comparing sequences obtained with the reference sequences from the known genotypes at each genetic locus.

### Sequence analysis

The secondary PCR products of the ITS, *tpi*, *bg* and *gdh* genes were sequenced in both directions on an ABI 3730 Genetic Analyzer (Applied Biosystems, Foster City, CA, USA). Nucleotide sequences generated were assembled and edited with software ChromasPro v.1.32 (http://technelysium.com.au/ChromasPro.html), and aligned with reference sequences from GenBank by using ClustalX (http://clustal.org).

### Phylogenetic analysis

To explore genetic diversity within the assemblage B of *G. duodenalis*, *tpi*, *bg* and *gdh* sequences from specimens with complete data at the three genetic loci were concatenated to form multi-locus sequences. They were compared with the reference sequences MLG1-MLG15 from NHPs, and Sweh001, Sweh059, Sweh074, Sweh107, Sweh136, Sweh158, ECUST5414, ECUST1710, ECUST4064 and ECUST981 from humans [28, 34, 51]. A maximum likelihood (ML) tree was constructed in MEGA v.6 (https://www.megasoftware.net) using evolutionary distances calculated by the commonly used general time reversible model. The reliability of clusters formed was assessed by bootstrap analysis using 1000 replicates.

### Statistical analysis

Differences in *E. bieneusi* and *G. duodenalis* detection rates between groups of different sex, age, or fecal consistency were assessed by using the Chi-square test implemented in SPSS Statistics v.20.0 (IBM Corp., Armonk, NY, USA). The difference was considered significant when *P* < 0.05.

## Results

### Occurrence of *E. bieneusi* and *G. duodenalis* in crab-eating macaques

Of the 1452 specimens analyzed, 461 (31.7%) were positive for *E. bieneusi*. Significantly higher detection rates of *E. bieneusi* were identified in animals with loose stools (41.4%, 151/365) than animals with normal stools (28.5%, 310/1087; *χ*^2^ = 20.83, *P* < 0.0001), in males (36.5%, 258/706) than females (26.7%, 160/599; *χ*^2^ = 14.391, *P* = 0.0001), and in old animals (> 3 years; 38.6%, 135/350) than young animals (1–3 years; 29.6%, 326/1102; *χ*^2^ = 9.90, *P* = 0.0016; Table 1).

**Table 1:** Distribution of *Giardia duodenalis* and *Enterocytozoon bieneusi* genotypes in crab-eating macaques in Hainan, China by fecal consistency, sex and age

Specimen	Sample size	*Giardia duodenalis* ^a^		*Enterocytozoon bieneusi*	
		No. positive (%)	Genotype (*n*)	No. positive (%)	Genotype (*n*)
Loose stools^b^	365	150 (41.1)	B (150)	151 (41.4)	Type IV (74)
					CM1 (40)
					Pongo2 (12)
					Peru8 (11)
					CM2 (8)
					Peru11 (4)
					PigEbITS7 (1)
					D (1)
Normal stools	1087	319 (29.3)	B (319)	310 (28.5)	Type IV (162)
					CM1 (79)
					Peru8 (31)
					Pongo2 (15)
					CM2 (9)
					Peru11 (8)
					CM3 (3)
					D (3)
Male^c^	706	236 (33.4)	B (236)	258 (36.5)	Type IV (134)
					CM1 (56)
					Peru8 (23)
					Pongo2 (18)
					CM2 (11)
					Peru11 (10)
					D (3)
					CM3 (2)
					PigEbITS7 (1)
Female	599	181 (30.2)	B (181)	160 (26.7)	Type IV (78)
					CM1 (53)
					Peru8 (14)
					Pongo2 (9)
					CM2 (5)
					D (1)
Unknown	147	52 (35.4)	B (52)	43 (29.3)	Type IV (24)
					CM1 (10)
					Peru8 (5)
					Peru11 (2)
					CM2 (1)
					CM3 (1)
1–3 years-old^d^	1102	403 (36.6)	B (403)	326 (29.6)	Type IV (160)
					CM1 (95)
					Peru8 (25)
					Pongo2 (17)
					CM2 (14)
					Peru11 (7)
					D (4)
					CM3 (3)
					PigEbITS7 (1)
> 3 years-old	350	66 (18.9)	B (66)	135 (38.6)	Type IV (76)
					CM1 (24)
					Peru8 (17)
					Pongo2 (10)
					Peru11 (5)
					CM2 (3)
Total	1452	469 (32.3)	B (469)	461 (31.7)	Type IV (236)
					CM1 (119)
					Peru8 (42)
					Pongo2 (27)
					CM2 (17)
					Peru11 (12)
					D (4)
					CM3 (3)
					PigEbITS7 (1)

^a^*Giardia duodenalis* detection rates were based on PCR analysis of the triosephosphate isomerase (*tpi*), glutamate dehydrogenase (*gdh*) and β-giardin (*bg*) genes

^b^Detection rates of *G. duodenalis* (*χ*^2^ = 17.25, *P* < 0.0001) and *E. bieneusi* (*χ*^2^ = 20.83, *P* < 0.0001) are significantly higher in animals with loose stools than in those with normal stools

^c^The detection rate of *E. bieneusi* is significant higher in males than in females (*χ*^2^ = 14.391, *P* = 0.0001)

^d^The detection rate of *G. duodenalis* is significantly higher in 1–3 year-old animals than in older animals (*χ*^2^ = 38.11, *P* < 0.0001). In contrast, the detection rate of *E. bieneusi* is significantly lower in 1–3 year-old animals than > 3 years (*χ*^2^ = 9.90, *P* = 0.0016)

For *G. duodenalis*, 362 (24.9%) specimens were positive by *tpi* PCR, 315 (21.7%) by *bg* PCR and 240 (16.5%) by *gdh* PCR. Altogether, 469 (32.3%) specimens were positive for *G. duodenalis* in at least one PCR. Significantly higher detection rates of *G. duodenalis* were found in animals with loose stools (41.1%, 150/365) than animals with normal stools (29.3%, 319/1087; *χ*^2^ = 17.25, *P* < 0.0001), and in 1–3 year-old monkeys (36.6%, 403/1102) than older animals (18.9%, 66/350; *χ*^2^ = 38.11, *P* < 0.0001). Nevertheless, detection rates of *G. duodenalis* were comparable between males (33.4%, 236/706) and females (31.2%, 233/746; Table 1).

### Distribution of *E. bieneusi* genotypes

Nine *E. bieneusi* genotypes were obtained from PCR-positive specimens by sequence analysis, namely Type IV (236/461), CM1 (119/461), Peru8 (42/461), Pongo2 (27/461), CM2 (17/461), Peru11 (12/461), D (4/461), CM3 (3/461) and PigEbITS7 (1/461).

Among them, eight *E. bieneusi* genotypes were found in animals with loose stools, namely Type IV (74/151), CM1 (40/151), Pongo2 (12/151), Peru8 (11/151), CM2 (8/151), Peru11 (4/151), PigEbITS7 (1/151) and D (1/151). Similarly, eight *E. bieneusi* genotypes were detected in animals with normal stools, namely Type IV (162/310), CM1 (79/310), Peru8 (31/310), Pongo2 (15/310), CM2 (9/310), Peru11 (8/310), CM3 (3/310) and D (3/310). A similar distribution of *E. bieneusi* genotypes was also seen between male and female monkeys as well as young and old monkeys (Table 1).

### Distribution of *G. duodenalis* genotypes and subtypes

Sequence analysis of PCR products from the *tpi*, *bg* and *gdh* genes showed that all 469 *G. duodenalis*-positive specimens had assemblage B (Table 1). Eight *G. duodenalis* subtypes were obtained from the 362 PCR-positive specimens at the *tpi* locus, including four known and four new subtypes. Among them, B-sh01 (*n* = 108), B1 (*n* = 75), B6 (*n* = 27) and B2 (*n* = 17) found in this study were identical to reference sequences JX994245, KC441076, GU564284 and KC441077, respectively. The new subtypes B-hn02 (*n* = 78), B-hn04 (*n* = 32), B-hn01 (*n* = 13) and B-hn03 (*n* = 12) had one, one, two and one single nucleotide polymorphism (SNP), respectively, compared with the reference sequence MF095053 (Table 2).

**Table 2:** Intra-genotypic nucleotide substitutions in the triosephosphate isomerase (*tpi*), glutamate dehydrogenase (*gdh*) and β-giardin (*bg*) genes of *Giardia duodenalis* in crab-eating macaques in Hainan, China

*tpi*													*gdh*									*bg*									
Subtype (*n*)	GenBank ID	Nucleotide at position											Subtype (*n*)	GenBank ID	Nucleotide at position							Subtype (*n*)	GenBank ID	Nucleotide at position							
Ref. sequence	MF095053	25	28	67	181	190	196	247	256	331	340	499	Ref. sequence	KM190707	297	561	666	681	786	793	876	Ref. sequence	KY696837	91	195	211	226	276	311	352	391
		A	T	C	G	T	A	G	C	G	G	G			C	T	T	C	C	G	G			A	A	T	T	A	A	C	A
B-sh01 (108)	JX994245												B-VANC/96/UBC/127 (162)	KM190707								B-CD10 (171)	KY696837								
B1 (75)	KC441076			T		C							B-VANC/87/UBC/8 (40)	KM190714		C				A		B2 (59)	KC441079	G			C			T	
B6 (27)	GU564284						G						B-VANC/91/UBC/67 (9)	KM190708					T	A		B-Egyh8 (58)	MG736242	G			C				
B2 (17)	KC441077	G	C					A					BIV (7)	KF679733		C						B-VANC/91/UBC/67 (5)	KM190799	G							
B-hn01 (13)^a^	MK262843				A						A		B-Afu97 (5)	HM134210		C		T		A		B-hn06^a^ (1)	MK282648					G	G		
B-hn02 (78)^a^	MK262844									A			B-sh03 (2)	JX994233	T		C					B-hn07^a^ (1)	MK282649		G						
B-hn03^a^ (12)	MK282645								T				B-hn05^a^ (15)	MK282647		C				A	A	B-hn08^a^ (20)	MK282650	G		G	C				G
B-hn04 (32)^a^	MK282646											A																			

^a^New subtype identified in this study

Seven *G. duodenalis* subtypes were present among the 315 PCR-positive specimens at the *bg* locus, including four known and three new subtypes. Among them, B-CD10 (*n* = 171), B2 (*n* = 59), B-Egyh8 (*n* = 58) and B-VANC/91/UBC/67 (*n* = 5) found in this study were identical to reference sequences KY696837, MG736242, KC441079 and KM190799, respectively. The new subtypes B-hn08 (*n* = 20), B-hn06 (*n* = 1) and B-hn07 (*n* = 1) had four, two, and one SNP, respectively, compared with the reference sequence KY696837 (Table 2).

Seven subtypes of *G. duodenalis* assemblage B were detected among the 240 PCR-positive specimens at the *gdh* locus, including six known ones and one new subtype. Among them, B-VANC/96/UBC/127 (*n* = 162), B-VANC/87/UBC/8 (*n* = 40), B-VANC/91/UBC/67 (*n* = 9), BIV (*n* = 7), B-Afu97 (*n* = 5) and B-sh03 (*n* = 2) found in this study were identical to the reference sequences KM190707, KM190714, KM190708, KF679733, HM134210 and JX994233, respectively. The new subtype B-hn05 (*n* = 15) had three SNPs compared with the reference sequence KM190707 (Table 2).

### Multilocus genotyping of assemblage B

Of the 469 specimens positive for *G. duodenalis* assemblage B, 161 were positive by PCR at all three genetic loci. They belonged to 53 MLGs (MLG-B-hn01 to MLG-B-hn53). Among them, MLG-B-hn01 (16.7%) was the most common, followed by MLG-B-hn02, MLG-B-hn03 and MLG-B-hn04, with frequencies of 7.5%, 6.2%, and 5.0%, respectively. In contrast, the frequency of MLG-B-hn05 and MLG-B-hn06 was 4.3%, the frequency of MLG-B-hn07 and MLG-B-hn08 was 3.7%, while the remaining MLGs were each seen in fewer than five specimens (Table 3).

**Table 3:** Multilocus sequence genotypes of *Giardia duodenalis* assemblage B in crab-eating macaques in Hainan, China

MLGs^a^	Subtype			No. of specimens
	*tpi*	*gdh*	*bg*	
MLG-B-hn01	B-sh01	B-VANC/96/UBC/127	B-CD10	27
MLG-B-hn02	B-hn02^b^	B-VANC/96/UBC/127	B-CD10	12
MLG-B-hn03	B1	B-VANC/96/UBC/127	B-Egyh8	10
MLG-B-hn04	B-hn03^b^	B-VANC/96/UBC/127	B-CD10	8
MLG-B-hn05	B1	B-VANC/96/UBC/127	B-CD10	7
MLG-B-hn06	B-sh01	B-VANC/87/UBC/8	B-CD10	7
MLG-B-hn07	B-hn02^b^	B-VANC/96/UBC/127	B2	6
MLG-B-hn08	B2	B-VANC/96/UBC/127	B-CD10	6
MLG-B-hn09	B-sh01	B-VANC/96/UBC/127	B2	4
MLG-B-hn10	B-hn02^b^	B-VANC/96/UBC/127	B-Egyh8	4
MLG-B-hn11	B1	B-VANC/96/UBC/127	B2	4
MLG-B-hn12	B-hn01^b^	B-VANC/96/UBC/127	B-CD10	3
MLG-B-hn13	B1	B-VANC/91/UBC/67	B-CD10	3
MLG-B-hn14	B1	B-hn05^b^	B-CD10	3
MLG-B-hn15	B6	B-VANC/96/UBC/127	B-CD10	3
MLG-B-hn16	B-sh01	B-VANC/87/UBC/8	B-Egyh8	3
MLG-B-hn17	B-sh01	B-VANC/87/UBC/8	B2	3
MLG-B-hn18	B1	B-VANC/87/UBC/8	B-CD10	2
MLG-B-hn19	B6	B-VANC/91/UBC/67	B-Egyh8	2
MLG-B-hn20	B-sh01	B-VANC/96/UBC/127	B-Egyh8	2
MLG-B-hn21	B-hn02^b^	BIV	B2	2
MLG-B-hn22	B-hn04^b^	B-VANC/96/UBC/127	B2	2
MLG-B-hn23	B-hn02^b^	B-hn05^b^	B-hn08^b^	2
MLG-B-hn24	B1	B-VANC/96/UBC/127	B-hn08^b^	2
MLG-B-hn25	B-sh01	B-VANC/91/UBC/67	B-CD10	2
MLG-B-hn26	B-sh01	B-hn05^b^	B-CD10	2
MLG-B-hn27	B1	B-VANC/87/UBC/8	B2	2
MLG-B-hn28	B1	B-VANC/87/UBC/8	B-hn08^b^	2
MLG-B-hn29	B-hn01^b^	B-VANC/87/UBC/8	B-CD10	2
MLG-B-hn30	B2	B-hn05^b^	B2	1
MLG-B-hn31	B1	B-sh03	B-CD10	1
MLG-B-hn32	B1	B-hn05^b^	B2	1
MLG-B-hn33	B-sh01	B-Afu97	B-CD10	1
MLG-B-hn34	B-hn02^b^	BIV	B-CD10	1
MLG-B-hn35	B6	B-hn05^b^	B-CD10	1
MLG-B-hn36	B-hn04^b^	B-VANC/91/UBC/67	B-CD10	1
MLG-B-hn37	B-hn04^b^	B-VANC/91/UBC/67	B-Egyh8	1
MLG-B-hn38	B1	B-VANC/87/UBC/8	B-Egyh8	1
MLG-B-hn39	B6	B-hn05^b^	B-Egyh8	1
MLG-B-hn40	B1	B-hn05^b^	B-Egyh8	1
MLG-B-hn41	B2	BIV	B2	1
MLG-B-hn42	B-sh01	B-hn05^b^	B2	1
MLG-B-hn43	B-hn04^b^	B-hn05^b^	B2	1
MLG-B-hn44	B-hn01^b^	B-VANC/96/UBC/127	B2	1
MLG-B-hn45	B-hn03^b^	B-VANC/96/UBC/127	B2	1
MLG-B-hn46	B-hn02^b^	B-VANC/87/UBC/8	B-hn08^b^	1
MLG-B-hn47	B-sh01	B-VANC/96/UBC/127	B-VANC/91/UBC/67	1
MLG-B-hn48	B6	B-Afu97	B-VANC/91/UBC/67	1
MLG-B-hn49	B2	B-VANC/96/UBC/127	B-hn08^b^	1
MLG-B-hn50	B-hn04^b^	B-VANC/87/UBC/8	B-CD10	1
MLG-B-hn51	B6	B-VANC/87/UBC/8	B-CD10	1
MLG-B-hn52	B1	B-VANC/87/UBC/8	B-VANC/91/UBC/67	1
MLG-B-hn53	B1	BIV	B-Egyh8	1

^a^MLGs are named based on subtypes at the *tpi*, *gdh* and *bg* loci

^b^New subtype identified in this study

### Phylogenetic relationship of *G. duodenalis* assemblage B

**Fig. 1:**
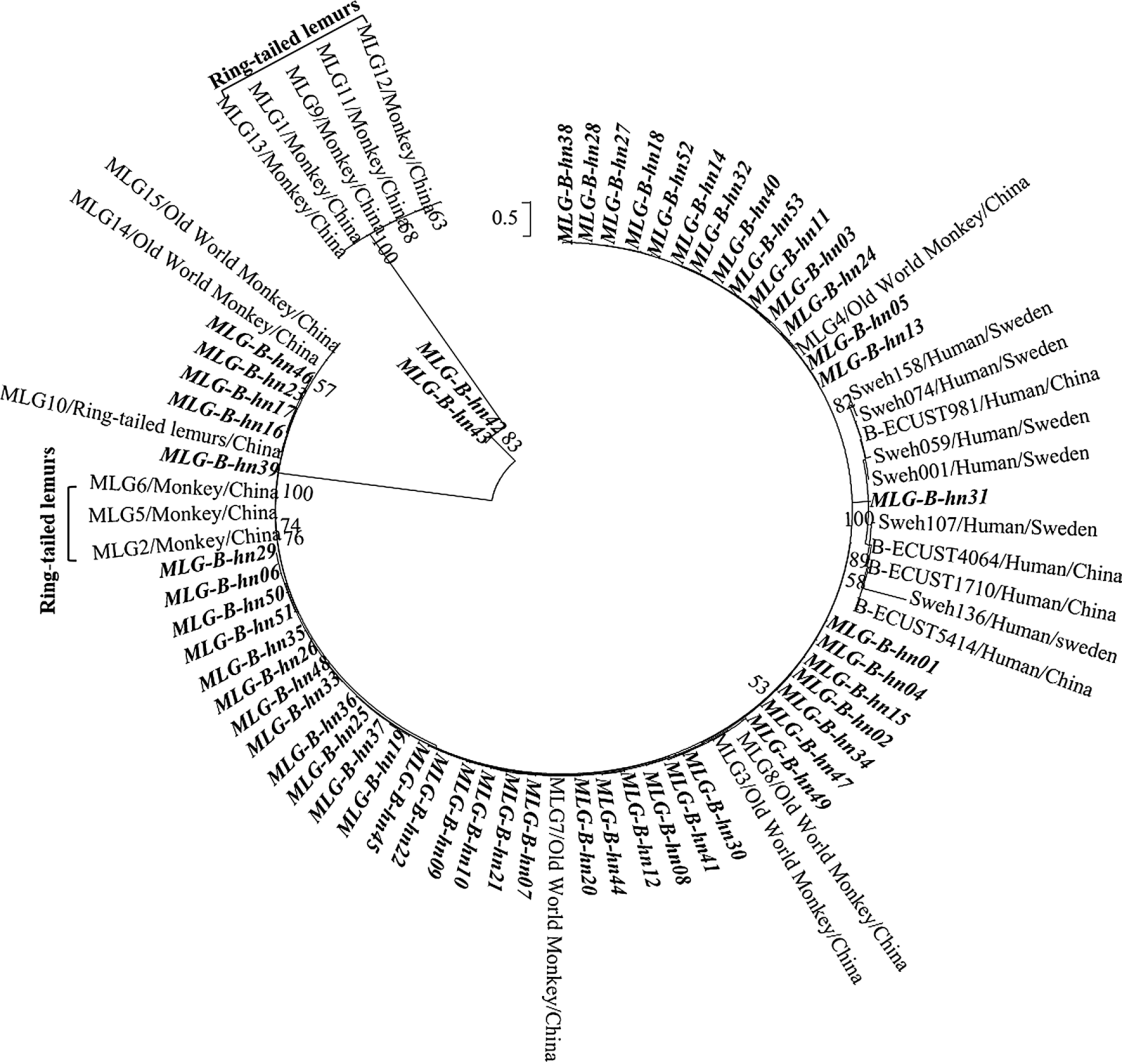
Phylogenetic relationship of multilocus genotypes (MLGs) of *Giardia duodenalis* assemblage B inferred by the maximum likelihood analysis of concatenated *tpi*, *gdh* and *bg* nucleotide sequences using genetic distances calculated by the general time reversible model (GTR). Reference sequences (MLG1-15, isolates Sweh001, Sweh059, Sweh074, Sweh107, Sweh136, Sweh158, ECUST1710, ECUST5414, ECUST4064 and ECUST981) used are from the studies by Lebbad et al. [51], Karim et al. [18] and Wang et al. [27]. Bootstrap values greater than 50% from 1000 replicates are shown on nodes. MLGs identified in the present study are in bold. The scale-bar indicates 50 nucleotide substitutions per 100 nucleotides

Phylogenetic analysis of concatenated sequences of the 53 assemblage B MLGs in this study, and those from previous studies [28, 34, 51] showed that most MLGs from this study were related to MLGs previously found in Old World monkeys (MLG-3, MLG-4, MLG-7, MLG-8, MLG-14 and MLG-15). However, one of the MLGs, MLG-B-hn31, seen in one animal, clustered together with MLGs in humans. In addition, MLG-B-hn42 and MLG-B-hn43 were genetically separated from Old World monkeys, ring-tailed lemurs and humans (Fig. 1).

## Discussion

Data from this study suggests that crab-eating macaques in Hainan, China are commonly infected with *E. bieneusi*. In this study, the detection rate of *E. bieneusi* in these animals was 31.7% (461/1452). This is higher than the reported detection rates in NHPs in various countries [52–55]. Similarly, it is mostly higher than detection rates in studies of *E. bieneusi* in NHPs in China [18, 19, 35, 50, 56–59]. Many of the studies reporting low detection rates of *E. bieneusi* in NHPs were performed using wild, captive and zoo animals [19, 35, 52, 54, 55, 57]. The present report represents the first one carried out using a large number of laboratory NHPs.

Crab-eating macaques are apparently infected with zoonotic *E. bieneusi* genotypes. In this study, all nine *E. bieneusi* genotypes detected in these animals belong to the zoonotic Group 1 (Table 1). Among them, Type IV, D, Peru8, Peru11 and PigEbITS7 are known human pathogens in many countries [13, 15, 26–29, 31, 60, 61]. Others such as CM1, CM2 and CM3 have been thus far only found in NHPs in China [18, 19, 62], but this is probably because only a small number of studies have been performed on human *E. bieneusi* infection within the country. The remaining genotype, Pongo2, was reported in China for the first time in this study. This genotype was initially seen in orangutans in Indonesia, indicating that it has the capability to infect a broad range of NHPs [53].

Laboratory crab-eating macaques are also apparently common hosts of *G. duodenalis*. In this study, the detection rate of *G. duodenalis* was 32.3% (469/1452) in Hainan, China. This confirms the prevalence of this pathogen in NHPs in various countries [36, 37, 63, 64] and different areas within China [34, 35, 38, 44, 50, 57, 65]. The very high detection rate of *G. duodenalis* as well as *E. bieneusi* in the present study could be attributed to the intensive farming of NHPs in this study, which congregates numerous susceptible individuals in confined areas.

To date, assemblages A, B and E of *G. duodenalis* have been reported in NHPs [34, 38, 50, 54, 65–67]. Among them, assemblage B is the most common genotype in different species of NHPs, including various monkeys, lemurs, gibbons, chimpanzees and gorillas [34, 36–38, 50, 57, 63–65]. It is also common in humans in both developing and industrialized countries, and is more common than the other major human-pathogenic genotype, assemblage A [2, 12, 28]. In this study, assemblage B was the only *G. duodenalis* genotype in the crab-eating macaque. This could have been due to the confined nature of animals in the facility, which limits the introduction of new *G. duodenalis* genotypes. Nevertheless, a high genetic heterogeneity of assemblage B was seen in animals in the laboratory facility, as revealed by subtype analysis at three genetic loci.

The zoonotic potential of *G. duodenalis* assemblage B in crab-eating macaques was supported by subtype analysis of specimens. Of the eight subtypes detected at the *tpi* locus, B-sh01 (JX994245) and B6 (GU564284) have been previously found in humans [28, 68]. Similarly, among the six known subtypes at the *gdh* locus, B-sh03 (JX994233) and B-VANC/87/UBC/8 (KM190714) have been previously found in humans [28, 69]. Likewise, among the four known *bg* subtypes, B-Egyh8 (MG736242) has been previously found in humans [69]. Therefore, many of the known subtypes of *G. duodenalis* obtained in this study at individual genetic loci had been previously found in humans, supporting the human-pathogenic potential of the assemblage B in crab-eating macaques.

Nevertheless, there appears to be some host segregation within assemblage B of *G. duodenalis* [34]. In this study, MLG analysis has identified 53 MLGs. Phylogenetic analysis showed that only MLG-B-hn31 is genetically similar to MLGs of assemblage B isolates from humans in China and Sweden [28, 51]. In contrast, most of other MLGs were genetically related to assemblage B isolates in pig-tailed macaques, rhesus macaques, golden monkeys, yellow baboons and green monkeys, all common Old-World monkeys. They were different from MLGs in ring-tailed lemurs, which are natives of the island nation Madagascar and evolved independently from monkeys and apes.

## Conclusions

In this study, we have shown a frequent occurrence and high genetic diversity *E. bieneusi* and *G. duodenalis* subtypes in crab-eating macaques in one commercial laboratory animal facility in Hainan, China. Most of the *E. bieneusi* genotypes and *G. duodenalis* assemblage B subtypes are potentially zoonotic. Additional genetic characterizations of these pathogens at other genetic loci, including more conservative ones for *G. duodenalis*, are needed to better understand the transmission of these pathogens and possible occurrence of host segregation within *G. duodenalis* assemblage B. Measures should be implemented at the commercial facility to reduce the transmission of enteric parasites.

## Data Availability

The data supporting the conclusions of this article are included within the article. Unique sequences generated in this study were submitted to the GenBank database under the accession numbers MK262843–MK262850.

## Abbreviations

PCR: polymerase chain reaction
MLG: multi-locus genotype
*bg*: beta-giardia
*gdh*: glutamate dehydrogenase
*tpi*: triosephosphate isomerase
ITS: internal transcribed spacer
